# 免疫相关不良事件在肺癌中发生和结局的真实世界研究

**DOI:** 10.3779/j.issn.1009-3419.2023.101.08

**Published:** 2023-04-20

**Authors:** CUI Shaohua, GE Xiaoxiao, LI Xiangyang

**Affiliations:** 200040 上海，复旦大学附属华东医院呼吸与危重症医学科; Department of Respiratory and Critical Care Medicine, Huadong Hospital, Fudan University, Shanghai 200040, China

**Keywords:** 肺肿瘤, 免疫相关不良事件, 免疫治疗, 生存期, Lung neoplasms, Immune-related adverse events, Immunotherapy, Survival time

## Abstract

**背景与目的** 免疫相关不良事件（immune-related adverse events, irAEs）常发生于免疫检查点抑制剂应用的患者，但关于肺癌中irAEs发生和结局的国内证据相对缺乏。本研究旨在评估中国肺癌患者接受免疫治疗后irAEs的发生和转归。**方法** 纳入2018年1月-2021年9月在复旦大学附属华东医院接受过至少1次免疫检查点抑制剂治疗的肺癌患者的临床和随访资料，通过统计描述、Kaplan-Meier等方法分析irAEs总体发生情况及各类irAEs的发生与结局。**结果** 共纳入135例患者，106例（78.5%）至少发生一种irAEs，首次发生的中位时间为28 d。多数irAEs发生于治疗早期，多为轻-中度、可恢复。57例（42.2%）患者死亡；严重irAEs致死率为12.6%（n=17），其中7例（41.2%）死于肺炎。整体人群的中位无进展生存期（progression-free survival, PFS）为505 d（95%CI: 352-658），中位总生存期（overall survival, OS）为625 d（95%CI: 491-759）。发生任一irAEs患者的PFS长于治疗未发生者（中位PFS分别为533 d和179 d，P=0.037；HR=0.57）；发生皮肤毒性者的OS长于未发生者（中位OS分别为797 d和469 d，P=0.006；HR=0.70）。**结论** 真实世界中肺癌患者irAEs普遍发生，其中肺炎为最常见的致死性irAEs，发生irAEs患者群体存在PFS上的优势。

## Abstract (en)

**Background and objective** Immune-related adverse events (irAEs) are commonly occurred in patients treated with immune checkpoint inhibitors. However, evidence of irAEs derived from the Chinese population is relatively lacking. The aim of this study was to investigate the incidence and outcomes of irAEs in Chinese patients with lung cancer after receiving immune checkpoint inhibitors (ICIs). **Methods** Clinical and follow-up data from lung cancer patients who received at least one time of ICIs from January 2018 to September 2021 at Huadong Hospital, Fudan University were included. Statistical descriptions and Kaplan-Meier method were used to analyze the overall incidence of irAEs, as well as the incidence and outcomes of each type of irAEs. **Results** 135 patients were included in the study. 106 patients (78.5%) presented at least one type of irAEs, and the median time to first irAEs onset was 28 d. Most irAEs occurred at early time after treatment, and most irAEs were mild-moderate and reversible. 57 patients (42.2%) died at the study cutoff. The mortality rate of severe irAEs was 12.6% (n=17), and among them 7 patients (41.2%) died of pneumonitis. The median progression-free survival (PFS) and overall survival (OS) time of the total population was 505 d (95%CI: 352-658) and 625 d (95%CI: 491-759), respectively. Patients who presented any irAEs achieved a longer PFS than those who did not (median PFS: 533 d vs 179 d, P=0.037, HR=0.57), while patients who presented skin toxicities achieved a longer OS than patients who did not (median OS: 797 d vs 469 d, P=0.006, HR=0.70). **Conclusion** In real-world settings, irAEs in lung cancer patients were commonly observed, with pneumonitis as the most common fatal irAEs. In addition, patients who presented any irAEs may tend to achieve a longer PFS.

**【Competing interests】** The authors declare that they have no competing interests.

免疫治疗是目前恶性肿瘤治疗的重要手段，以针对程序性死亡受体1（programmed cell death 1, PD-1）或其配体（programmed cell death ligand 1, PD-L1）信号通路为代表的免疫检查点抑制剂，对包括肺癌在内的多种实体瘤都具有良好的抗肿瘤效应。与化疗相比，免疫治疗能明显改善晚期肿瘤患者的生存结局，或延长可手术患者的术后无病生存期^[1]^。KEYNOTE-024研究^[2]^表明，帕博利珠单抗较化疗能显著提高PD-L1高表达晚期初治非小细胞肺癌患者的无进展生存期（progression-free survival, PFS）和总生存期（overall survival, OS）；IMpower010研究^[3]^的数据也证实辅助化疗后进行阿替利珠单抗辅助免疫治疗可显著改善II期-IIIA期非小细胞肺癌患者的无病生存期。

免疫治疗疗效虽好，但安全性值得关注。免疫检查点抑制剂在通过激活体内免疫系统发挥抗肿瘤作用的同时，也会导致一系列毒性事件，即免疫相关不良事件（immune-related adverse events, irAEs）^[4,5]^。irAEs的发生可能与针对共同抗原的T细胞激活、体内预先存在自身抗原水平升高、促炎细胞因子水平增高等因素有关^[4]^。临床试验数据^[6⇓-8]^表明，irAEs发生非常常见，多数接受免疫检查点抑制剂治疗的受试者会出现任一级别irAEs，而约10%受试者会出现3级及以上irAEs。这些irAEs会涉及体内诸多系统，包括皮肤、胃肠道、内分泌系统、肺、肝脏等。irAEs发生的复杂性为临床免疫治疗带来挑战。其发生类型和特征与免疫检查点抑制剂的种类和患者自身状态有关；而某种irAEs发生的严重程度，也可从无症状，到严重威胁生命^[9]^。此外，某些irAEs的发生存在延迟效应，可在治疗后很长时间甚至停药后发生^[10,11]^。由此可见，临床随访过程中对irAEs的监测和及时干预至关重要。美国临床肿瘤协会、美国国立综合癌症网络、中国临床肿瘤学会等学术组织针对irAEs的治疗指南，对各类irAEs的处理措施进行了推荐^[12⇓⇓-15]^。

然而，目前对irAEs发生模式的认识及治疗指南的制定，多数基于国外临床试验数据，国内证据相对缺乏。此外，与纳入排除标准限制极其严格的临床试验相比，真实世界临床实践中的情况更为复杂，临床试验结果难以反映临床实践中的情况。基于此，我们开展这项单中心、真实世界观察性研究，评估免疫检查点抑制剂单药或联合治疗肺癌后发生irAEs的情况及结局，为中国肺癌患者提供免疫治疗安全性方面的研究证据。

## 1 资料与方法

### 1.1 研究对象

纳入2018年1月-2021年9月在复旦大学附属华东医院接受过至少1次PD-1/PD-L1抗体进行免疫治疗的肺癌病例。（1）纳入标准：经组织学或细胞学明确的肺癌患者，包括非小细胞肺癌和小细胞肺癌；年龄≥18岁；免疫检查点抑制剂作为单药或联合其他全身抗肿瘤治疗；免疫检查点抑制剂可作为围手术期治疗，或针对晚期疾病的姑息性治疗。（2）排除标准：多部位原发恶性肿瘤；首次接受免疫检查点抑制剂治疗14 d内接受系统性类固醇激素（泼尼松：等效剂量>10 mg/d）、其他抑制免疫的药物；曾接受过抗肿瘤疫苗或免疫刺激药物（包括先前曾使用过免疫检查点抑制剂）；病例基线资料不全，以及每例患者定期随访期间存在各类irAEs数据不完整者。

本项目的研究方案已通过复旦大学附属华东医院伦理委员会审批（No.20220100）。

### 1.2 资料收集

通过查阅电子病历收集患者基线和临床病理资料，包括：性别、年龄、肿瘤类型、治疗前临床分期、免疫治疗情况等。irAEs分类：根据《免疫检查点抑制剂相关的毒性管理指南》^[15]^，将irAEs分为皮肤毒性、胃肠毒性、内分泌毒性、肺毒性、肝脏毒性、神经系统毒性、血液毒性、肾脏毒性、心脏毒性、眼毒性等。irAEs分级：参照《不良事件通用术语标准（Common Terminology Criteria for Adverse Events, CTCAE）》5.0版，通过查阅住院电子病历、电话随访等途径，收集、记录各类irAEs发生情况，并记录irAEs是否影响免疫治疗、是否恢复等。采用实体瘤疗效评价标准（Response Evaluation Criteria in Solid Tumors, RECIST）1.1版进行治疗疗效评估。记录疾病进展情况和生存状况。研究截止日期为2022年12月31日。

### 1.3 时间-事件数据的定义

时间-事件数据定义：irAEs发生时间定义为自使用免疫治疗至首次发生irAEs的时间。PFS定义为自接受免疫治疗起至根据RECIST 1.1评估的疾病复发、进展或死亡的时间；OS定义为自接受免疫治疗起至死亡的时间。研究截止日期时未达到终点的事件按截尾数据处理。

### 1.4 统计学分析和软件使用情况

（1）统计描述：计数资料采用“例数（构成比）”描述，计量资料采用“中位数（范围）”描述。（2）假设检验：采用Kaplan-Meier法估算各类irAEs发生中位时间、中位PFS及OS；采用Log-rank检验比较组间PFS和OS；采用Cox回归（Enter法）估计风险比（hazard ratio, HR）。（3）统计软件及统计学规定：采用SPSS 24.0版进行统计分析，双侧P<0.05为差异具有统计学意义。

## 2 结果

### 2.1 病例人口学基线和临床病理特征

纳入135例肺癌患者的人口学及临床病理特征见表1。研究队列的中位年龄为66岁（最小37岁，最大83岁），多数为男性（108例，80.0%）、具有吸烟史（86例，63.7%）、体力状况评分（performance status, PS）为0分-1分（112例，83.0%）。病理亚型为肺腺癌、鳞状细胞癌、小细胞肺癌患者分别为80例（59.3%）、33例（24.4%）和13例（9.6%）；临床分期多为IIIB期-IV期（121例，89.6%）。

**表1:** 接受ICIs治疗的135例肺癌患者的临床特征

Clinical characteristics		n	Percentage (%)
Age [median (range)] (yr)		66 (37-83)	
Gender	Male	108	80.0
	Female	27	20.0
Smoking status (smoking index)	Never	49	36.3
	200-400	7	5.2
	>400	79	58.5
PS	0-1	112	83.0
	2-3	23	17.0
Histology	Adenocarcinoma	80	59.3
	Squamous cell carcinoma	33	24.4
	Other non-small cell lung cancer	9	6.7
	Small cell lung cancer	13	9.6
PD-L1 TPS	Negative	35	25.9
	<1%	5	3.7
	1%-50%	23	17.0
	>50%	23	17.0
	Unknown	49	36.3
TNM stage	IB-IIIA	14	10.4
	IIIB-IV	121	89.6
Antibodies	Anti PD-1	125	92.6
	Anti PD-L1	10	7.4
Treatment	Neoadjuvant	12	8.9
	Adjuvant	2	1.5
	First-line	69	51.1
	Second-line	32	23.7
	Third-line	20	14.8
Combination	None	23	17.0
	Chemotherapy	96	71.1
	Other	16	11.9

Smoking index: number of cigarettes smoked per day multiply by years of smoking. ICIs: immune checkpoint inhibitors; PS: performance status; PD-L1: programmed cell death ligand 1; PD-1: programmed cell death 1; TPS: tumor proportion score; TNM: tumor-node-metastasis.

免疫检查点抑制剂治疗方面，多数患者采用抗PD-1治疗（125例，92.6%）；联合化疗者为96例（71.1%），其中含铂双药联合92例（培美曲塞、多西他赛、吉西他滨或依托泊苷联合卡铂/顺铂/奈达铂），单药联合4例（均为联合培美曲塞）。

### 2.2 总体irAEs发生情况

本研究的中位随访时间为443 d（95%CI: 380-506）。irAEs整体发生情况见表2。106例（78.5%）患者发生至少一种irAEs，其中首个irAEs发生的中位时间为治疗后28 d（95%CI: 24-32），首个发生的irAEs多为皮肤毒性（44例，32.6%）、骨髓抑制（14例，10.4%）和肝毒性（12例，8.9%）。研究过程中1个、2个及3个器官（或系统）受累的病例分别为46例（34.1%）、37例（27.4%）及16例（11.9%）。

**表 2:** 135例肺癌患者发生免疫相关不良事件的总体情况

Information		n (%)
Time to first irAEs onset [median (95%CI)] (d)		28 (95%CI: 24-32)
Patients with any irAEs onset		106 (78.5)
Total number of organs being affected	0	29 (21.5)
	1	46 (34.1)
	2	37 (27.4)
	3	16 (11.9)
	4	6 (4.4)
	5	1 (0.7)
Toxicities being first occured	Skin toxicities	44 (32.6)
	Myelosuppression	14 (10.4)
	Hepatitis	12 (8.9)
	Gastrointestinal toxicities	10 (7.4)
	Pneumonitis	8 (5.9)
	Endocrine toxicities	8 (5.9)
	Other toxicities	10 (7.4)

CI: confidence interval; irAEs: immune-related adverse events.

### 2.3 各类irAEs发生及结局

各器官irAEs发生及结局见表3。由表可见，多数器官irAEs出现于治疗早期。

**表3:** 各器官免疫相关不良事件发生及转归情况

irAEs categories		Severity, n (%)		Median onset time (range) (d)	Recovery [n (%)]	irAEs leading to ICIs adjustment [n (%)]	irAEs leading to death [n (%)]
		Any grade	Grade 3-5				
Skin toxicities	Rash	24 (17.8)	1 (0.7)	26 (22-30)	23 (95.8)	6 (25.0)	0 (0.0)
	Telangiectasia	23 (17.0)	2 (1.5)	34 (28-40)	19 (82.6)	8 (34.8)	0 (0.0)
	Pruritus	14 (10.4)	0 (0.0)	36 (34-38)	11 (78.6)	1 (7.1)	0 (0.0)
	Vitiligo	2 (1.5)	1 (0.7)	14 (NA)	1 (50.0)	1 (50.0)	0 (0.0)
Myelosuppression		27 (20.0)	12 (8.9)	35 (24-46)	24 (88.9)	11 (40.7)	1 (3.7)
Hepatitis		29 (21.5)	3 (2.2)	57 (39-75)	29 (100.0)	9 (31.0)	0 (0.0)
Gastrointestinal toxicities	Gastritis	7 (5.2)	2 (1.5)	12 (9-15)	6 (85.7)	4 (57.1)	1 (14.3)
	Colitis	8 (5.9)	5 (3.7)	20 (11-29)	5 (62.5)	6 (75.0)	3 (37.5)
Pneumonitis		25 (18.5)	11 (8.1)	88 (42-134)	16 (64.0)	16 (64.0)	7 (28.0)
Endocrine toxicities	Hyperthyroidism	5 (3.7)	0 (0.0)	96 (0-259)	4 (80.0)	0 (0.0)	0 (0.0)
	Hypothyroidism	13 (9.6) *	0 (0.0)	153 (74-299)	10 (76.9)	1 (7.7)	0 (0.0)
	Hypophysitis	3 (2.2)	3 (2.2)	127 (0-305)	2 (66.7)	3 (100.0)	1 (33.3)
Renal insufficiency		9 (6.7)	1 (0.7)	43 (23-63)	6 (66.7)	5 (55.6)	0 (0.0)
Myocarditis		5 (3.7)	4 (3.0)	63 (50-76)	4 (80.0)	5 (100.0)	4 (80.0)
Uveitis		1 (0.7)	0 (0.0)	48 (NA)	1 (100.0)	0 (0.0)	0 (0.0)
Pulmonary embolism		1 (0.7)	1 (0.7)	30 (NA)	0 (0.0)	1 (100.0)	1 (100.0)
Arthritis		2 (1.5)	1 (0.7)	31 (NA)	1 (50.0)	0 (0.0)	0 (0.0)
Nerve toxicities	Encephalitis	1 (0.7)	1 (0.7)	58 (NA)	0 (0.0)	1 (100.0)	1 (100.0)
	Peripheral neuritis	4 (3.0)	0 (0.0)	62 (2-128)	3 (75.0)	0 (0.0)	0 (0.0)

*One case transferred from hyperthyroidism. NA: not applicable.

皮肤毒性和内分泌毒性可有多种临床表现。其中，皮肤毒性中，皮疹（24例，17.8%）、毛细血管扩张（23例，17.0%）最常见，中位发生时间分别为治疗后26 d、34 d。

内分泌毒性可表现为甲亢（5例，3.7%）、甲减（13例，9.6%）和垂体炎（3例，2.2%），其中1例患者由甲亢转化为甲减。

此外，发生骨髓抑制、肝毒性、肺炎的患者分别为27例（20.0%）、29例（21.5%）、25例（18.5%）。

irAEs多为轻-中度，多数可经免疫治疗剂量调整及对症治疗后恢复。研究截止时，死亡病例57例（42.2%），其中17例死于严重irAEs，irAEs致死率为12.6%，这些患者包括肺炎（7例）、心肌炎（4例，其中1例合并肺炎）、肠炎（3例）、骨髓抑制（1例）、垂体炎（1例）、肺栓塞（1例）、脑炎（1例）。

### 2.4 irAEs发生与PFS和OS的关系

截至研究结束，队列发生疾病进展66例（66/133, 49.6%），死亡57例（57/135, 42.2%）。整体人群的中位PFS为505 d（95%CI: 352-658），中位OS为625 d（95%CI: 491-759）。

对任一irAEs是否发生以及发生率超过15%的各器官irAEs是否发生与PFS及OS进行相关性分析发现，发生任一irAEs患者的PFS明显长于治疗随访过程中未发生irAEs的患者，中位PFS分别为533 d（95%CI: 395-671）和179 d（95%CI: 0-455）（P=0.037; HR=0.57, 95%CI: 0.33-0.97）（表4，图1）；发生皮肤毒性患者的OS明显长于未发生皮肤毒性的患者，中位OS分别为797 d（95%CI: 520-1,074）和469 d（95%CI: 229-709）（P=0.006; HR=0.70, 95%CI: 0.51-0.90）（表4，图2）。其他比较中发现组间差异无统计学意义（表4）。

**表4:** irAEs是否发生与PFS及OS的关系

irAEs categories	PFS (n=133)				OS (n=135)		
	Patients with irAEs vs patients without irAEs [Median (95%CI)] (d)	P	HR (95%CI)		Patients with irAEs vs patients without irAEs [Median (95%CI)] (d)	P	HR (95%CI)
Any irAEs	533 (395-671) vs 179 (0-455)	0.037*	0.57 (0.33-0.97)		625 (511-739) vs 443 (55-831)	0.178	0.67 (0.37-1.21)
Skin toxicities	562 (401-723) vs 342 (48-636)	0.101	0.81 (0.63-1.04)		797 (520-1,074) vs 469 (229-709)	0.006*	0.70 (0.51-0.90)
Myelosuppression	NR vs 501 (344-658)	0.052	0.71 (0.50-1.01)		NR vs 600 (458-742)	0.260	0.82 (0.57-1.17)
Hepatitis	364 (111-617) vs 505 (321-689)	0.371	0.87 (0.64-1.18)		625 (340-910) vs 600 (459-741)	0.771	0.95 (0.69-1.31)
Endocrine toxicities	533 (256-810) vs 505 (313-697)	0.435	0.87 (0.61-1.24)		625 (575-675) vs 556 (351-761)	0.081	0.67 (0.42-1.06)
Pneumonitis	603 (156-1050) vs 501 (338-664)	0.527	0.90 (0.66-1.24)		560 (309-811) vs 625 (488-762)	0.817	0.96 (0.69-1.34)

PFS: progression-free survival; OS: overall survival; HR: hazard ratio; NR: not reached. *P<0.05. 2 data missing for PFS.

**图 1:**
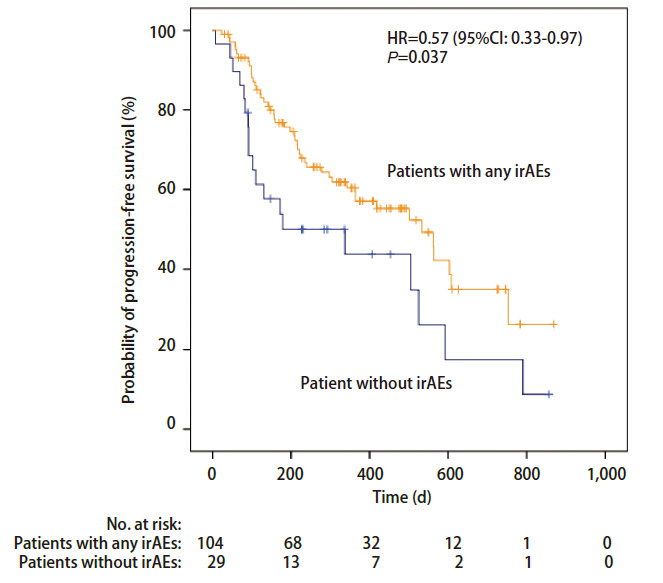
采用Kaplan-Meier法绘制的发生任一irAEs患者和未发生irAEs患者群体的PFS曲线

**图 2:**
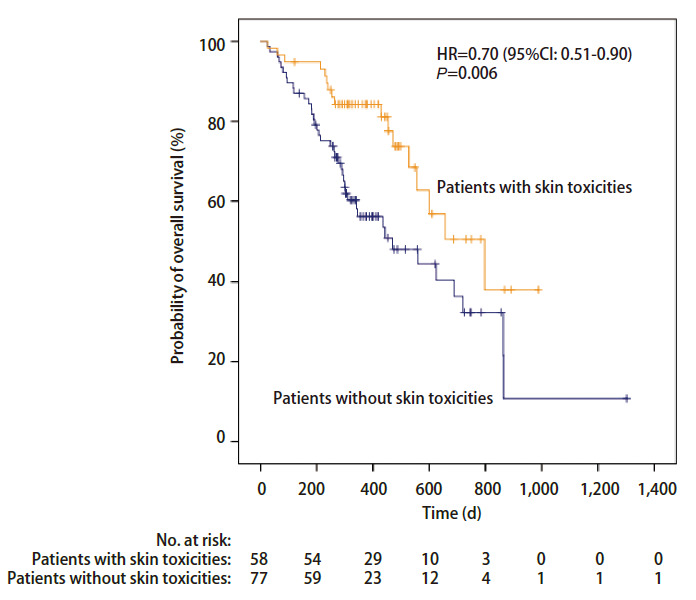
采用Kaplan-Meier法绘制的发生皮肤毒性患者和未发生皮肤毒性患者群体的OS曲线

## 3 讨论

这项单中心真实世界观察性研究，纳入135例接受免疫检查点抑制剂单药或联合其他治疗的肺癌患者，系统评估治疗后irAEs发生状况及转归情况，并对irAEs是否发生与生存结局PFS、OS的关系进行探讨。真实世界临床实践中，肺癌患者接受免疫治疗后发生irAEs非常普遍，多数irAEs发生于治疗早期且可恢复，但因为我们观察到部分irAEs（如肺炎等）可在治疗较晚阶段出现且危及生命，irAEs密切监测和及时干预至关重要。

我们的研究提示，irAEs总体发生率高达78.5%，这与既往研究^[16]^报道一致。irAEs可影响到全身多个系统和/或器官，引起一系列临床表现。我们的研究中，皮肤毒性是最早出现也是最常见的irAEs，其临床多为皮疹、毛细血管扩张、瘙痒、白癜风，中位发生时间约在免疫治疗后1个月，其中毛细血管扩张主要表现为血管瘤。皮肤毒性多数能缓解，部分需要免疫治疗减量或延期后缓解。此外，骨髓抑制和肝毒性也是本研究发现的常见不良事件。71.1%患者在免疫治疗时联合应用了化疗，因此骨髓抑制和肝毒性发生率可能会较单纯进行免疫治疗的队列增加^[17]^。这部分患者在临床随访中应规律监测血常规及肝功能，必要时应增加随诊次数。

免疫检查点抑制剂相关肺炎也是免疫治疗后相对常见的irAEs，其发生率和致死率在不同研究中有差异^[18⇓-20]^。一项meta分析^[21]^提示非小细胞肺癌患者发生免疫检查点抑制剂相关肺炎的可能性较其他肿瘤更大。值得注意的是，我们的真实世界队列中肺炎发生率接近20%，且是irAEs导致患者死亡的首要原因。真实世界临床实践中患者情况相较临床试验更为复杂，免疫检查点抑制剂相关肺炎的发生率和致死率可能更高，提示在临床随诊中应格外重视。对于高龄、既往吸烟史、存在肺部疾病、曾接受胸部放疗的患者，其发生免疫检查点抑制剂相关肺炎的风险相对更高^[22]^，在进行免疫治疗，特别是联合治疗时应格外注意。接受免疫检查点抑制剂治疗后发生肺炎还可能与免疫检查点抑制剂的类别有关，如接受PD-1/PD-L1抑制剂治疗患者的肺炎发生率较接受细胞毒T淋巴细胞相关抗原4（cytotoxic T lymphocyte-associated antigen-4, CTLA-4）抑制剂患者肺炎发生率更高^[23]^。 除常规肿瘤评估时间点进行胸部计算机断层扫描（computed tomography, CT）检查外，如患者出现咳嗽、胸闷、呼吸困难等症状时，必要时应进行肺炎排查，特别是在免疫治疗后2个月-3个月。此外，临床医生还应注意与其他病因引起的肺炎相鉴别。

内分泌系统也是irAEs常见的发生部位，本研究中监测到的内分泌相关irAEs包括甲状腺毒性（包括甲亢、甲减）和垂体炎，发生的中位时间约在免疫治疗开始后的3个月-5个月，因此对甲状腺等内分泌功能的常规检查也是必要的。除以上这些常见的不良事件外，我们还观察到一些相对少见的irAEs，包括心肌炎、葡萄膜炎、肺栓塞、脑炎、关节炎等，这些irAEs虽然少见，但部分很严重，甚至危及生命，提示在随访过程中必须引起足够重视。

总体上，本研究出现irAEs的时间和irAEs发生率较既往研究更高。例如，胃肠毒性一般发生于治疗后5周-8周^[24,25]^，而我们研究中中位发生时间为3周-4周。其原因主要与两点因素有关。第一，与随机对照临床研究相比，真实世界群体异质性更大，接受治疗患者范围相对更广；第二，本研究多数患者在进行免疫检查点抑制剂治疗的基础上联合应用化疗等，这些药物的应用也会潜在促进irAEs发生，使irAEs发生率更高和出现更早。

有研究^[26⇓-28]^发现irAEs发生与患者预后有关。本研究对整体irAEs及常见的几类irAEs与患者生存结局PFS及OS关系的探讨中发现，与不发生任何irAEs的患者相比，发生任一irAEs的患者PFS更长，疾病进展或死亡风险下降43%；此外，我们发现，与不发生皮肤毒性的患者相比，发生皮肤毒性患者的OS更长，死亡风险下降30%。提示肺癌患者进行免疫检查点抑制剂治疗的生存结局可能与irAEs发生存在某种关系。近期的一项研究^[29]^表明，发生irAEs患者会存在PFS和OS获益。我们的研究虽没有得到发生irAEs患者OS获益的统计学差异，但在数值上存在这一趋势。此外，另外一项研究^[30]^表明，irAEs发生的严重程度也与患者PFS获益有关，研究者发现发生3级及以上irAEs的患者更可能出现PFS获益。

本研究存在一些不足之处。作为单中心研究，纳入的总病例数和随访时间均相对有限。此外，对非小细胞肺癌、小细胞肺癌等亚组分析，也因病例数较少难以开展。研究结果有待今后开展更大样本的前瞻性研究证实。但作为一项在中国人群中开展的真实世界研究，我们的结果提示，irAEs的发生是普遍的，潜在受到影响的器官多，且部分irAEs有威胁到生命的风险，需要临床医生在关注免疫治疗疗效的同时关注其可能出现的不良事件，这为临床肺癌患者应用免疫检查点抑制剂提供了安全性方面的证据。
